# A New Real-Time Pinch Detection Algorithm Based on Model Reference Kalman Prediction and SRMS for Electric Adjustable Desk

**DOI:** 10.3390/s20174699

**Published:** 2020-08-20

**Authors:** Minming Gu, Yajie Wei, Haipeng Pan, Yujia Ying

**Affiliations:** 1 Faculty of Mechanical Engineering & Automation, Zhejiang Sci-Tech University, Hangzhou 310018, China; guminming@zstu.edu.cn (M.G.); 2018g0507020@mails.zstu.edu.cn (Y.W.); 2 Nanhu College, Jiaxing University, Jiaxing 314001, China; yyj@zjxu.edu.cn

**Keywords:** anti-pinch detection, model reference adaptive kalman prediction, sliding root mean square (SRMS), height adjustable desk

## Abstract

This paper presents a new algorithm based on model reference Kalman torque prediction algorithm combined with the sliding root mean square (SRMS). It is necessary to improve the accuracy and reliability of the pinch detection for avoiding collision with the height adjustable desk and accidents on users. Motors need to regulate their position and speed during the operation using different voltage by PWM (Pulse Width Modulation) to meet the requirement of position synchronization. It causes much noise and coupling information in the current sampling signal. Firstly, to analyze the working principle of an electric height adjustable desk control system, a system model is established with consideration of the DC (Direct Current) motor characteristics and the coupling of the system. Secondly, to precisely identify the load situation, a new model reference Kalman perdition method is proposed. The load torque signal is selected as a pinch state variable of the filter by comparing the current signal. Thirdly, to meet the need of the different loads of the electric table, the sliding root means square (SRMS) of the torque is proposed to be the criterion for threshold detection. Finally, to verify the effectiveness of the algorithm, the experiments are carried out in the actual system. Experimental results show that the algorithm proposed in this paper can detect the pinched state accurately under different load conditions.

## 1. Introduction

People spend quite a large portion of their day-time in sedentary behavior [1]. With the continual increase of sitting down hours, musculoskeletal system problems are becoming more and more serious [2]. For the sake of alleviating this problem, the electric height adjustable desk is widely used in today’s office worldwide [3]. Automatically adjusting the height of the desk using DC (Direct Current) motors provides an alternative working pattern to choose standing or sitting. Meanwhile, it also brings some safety problems during its rising or falling. Children or careless people may injure themselves when they misuse a height adjustable desk. Thus, during the movement, the state must be monitored and diagnosed effectively to guarantee safe operation and prevent human casualties. Therefore, anti-pinch detection is an essential function in the adjustable height desk and plays a crucial role in reliable operation.

Many researchers have conducted intensive research on anti-pinch detection. In pinch detection of car window, which is similar to the height adjustable desk, angular velocity detection [4,5], armature current detection [6,7,8,9], torque rate detection [10,11,12], and torque detection [13,14,15] of the motor are often used. As the armature current value and motor torque of DC motor are reflected in the running state, those threshold values are selected as a basis for judgment [7]. When the desk meets obstacles during the rising or falling process, current or torque value exceeds the threshold, and the window begins to retract. Unfortunately, it has a poor performance in the real system. As the desk should keep the position synchronization of different desk legs, the cross-coupling between motors [16] will be obvious. Meanwhile, compared with windows, the PWM (Pulse Width Modulation) signal of motor speed regulation will also introduce noise. To make up for the inadequateness in the practical situation, choosing the appropriate filtering algorithm is the key.

There exist various kinds of filter design, such as H∞ filter [17,18], H_-/H_∞ filter [19], Kalman filter [20,21], particle filters [22,23], fast Fourier transform (FFT) [24,25], wavelet transform (WT) [26,27,28,29], empirical mode decomposition (EMD) [30,31], independent component analysis (ICA) [32], and so on. H∞ filter does not make an assumption about the noise, and it minimizes the maximum estimation error, but H∞ filter cannot guarantee that the pinch system’s estimation error is minimized owing to its high computational cost [33]. Particle filtering shows obvious superiority in processing the nonlinear state-space model [34]. Particle filtering uses Monte Carlo simulation to achieve recursive Bayesian filter by a large number of random samples called particles. The core is to use a group of random samples (particles) with corresponding weights to represent the posterior distribution of states, and the weighted sum of random samples is used as estimates of states. Filter design lacks the analysis of the input interference signals, which easily leads to deviations and divergences in the estimates. Owing to its multi-resolution properties and behavior of singularities [29], wavelet transform can locate the particular points and widely used in many domains. However, wavelet transform needs a high operating cost for real-time detection. For electric height adjustable desk pinch detection, the filter method not only should be intelligible to be compatible with different load conditions, but should also have low computational costs. Among these algorithms, the Kalman filter has a lower calculation and is suitable for real-time detection. The Kalman filter is mainly used to estimate system states that can only be observed indirectly or inaccurately by the system itself [35].

On the other hand, the fixed absolute pinch estimator used in the car window [13] is not suitable for our plant. It does have a fast response and is easy to implement. However, the load over the height adjustable desk is uncertain and would be changed anytime. The fixed value cannot distinguish the actual load situation. It might not be a general solution because the plants would easily miss the pinch and make false alarms. A variable threshold will be able to distinguish the situation of the pinch better.

In this paper, the electric height adjustable desk system, which uses sampling resistors as the current detection sensor of the motor, has adopted a new real-time pinch detection algorithm based on model reference Kalman prediction and dynamic threshold using sliding root mean square (SRMS). In this algorithm, the mathematical and simulation model is established to present the relationship between the DC motor load torque and input variables, which contains current signals, voltage, and noises. Moreover, the armature voltage is given by the based on the cross-coupling control. Then, the load torque estimation based on the steady-state Kalman filter is presented. The motor parameter identification [36] is conducted to improve the performance of the steady-state Kalman filter. The SRMS of pinch torque is used as the anti-pinch criterion, which not only quickly identifies the anti-pinch points of different load states, but also has strong robustness under different working conditions. 

## 2. Model of Electrical Height Adjustable Desk

The electric height adjustable desk is commonly driven by two permanent magnets brushed DC motors (PMDC). For a DC motor drive system, there are two essential balance equations, including voltage and torque balance equation in model. The voltage balance equation can be expressed as follows:

(1a) $$L\frac{dI_{d}}{dt}=U_{d}-I_{d}R-E_{a}$$

(1b) $$E_{a}=K_{e}*\omega$$

The torque balance equation can be expressed as follows:

(2a) $$\begin{array}{c} J\frac{d\omega }{dt}=T_{c}-T_{l}-T_{\mu }+\mu_{v} \end{array}$$

(2b) $$\begin{array}{c} T_{c}=K_{t}*I_{d} \end{array}$$

(2c) $$\begin{array}{c} T_{l}=T_{load}+T_{p} \end{array}$$

(2d) $$\begin{array}{c} T_{\mu }=B_{\mu }*\omega \end{array}$$

In Equation (2c), $T_{l}$ is subdivided into $T_{load}$ and $T_{p}$. $\;T_{load}$ is the load torque, which is used to drive load in a normal condition of an electric height adjustable desk. $T_{load}$ is proportional to the weight of the load. $\;T_{p}$ is the pinch torque that only appears when obstacles hinder the raising and lowering of the height adjustable desk. As the vibration torque $\mu_{v}$ varies during its operating condition, it can be assumed as a zero-mean white noise input $u_{\mu_{v}}$ with the variance $Q_{\mu_{v}}$.

On the basis of the above balance equations, $I_{d}$ and ω are selected as the state variables. The state-space model of DC motor can be expressed as follows:

(3) $$\begin{array}{c} \{\begin{array}{c} \dot{x}=Ax+Bu+w\;\\ y=Cx+v \end{array} \end{array}$$

where

$$\begin{array}{c} A=\left[\begin{array}{cc} -\frac{R}{L} & -\frac{K_{e}}{L}\\ \frac{K_{t}}{J} & -\frac{B_{\mu }}{J} \end{array}\right],\;B={\left[\frac{1}{L}\;-\frac{1}{J}\right]}^{T},\;C={\left[1\;0\right]}^{T}\\ x={\left[I_{d}\;\omega \right]}^{T},\;u={\left[U_{d}\;T_{l}\right]}^{T},\;w={\left[0\;\mu_{v}\right]}^{T} \end{array}$$

Here, w is the plant noise of the height adjustable desk system and v is the measurement noise of a height adjustable desk system. $U_{d}$ is armature voltage, which is a time variable. It is the output of the following controller, which contains the PWM signal.

**Figure 1:**
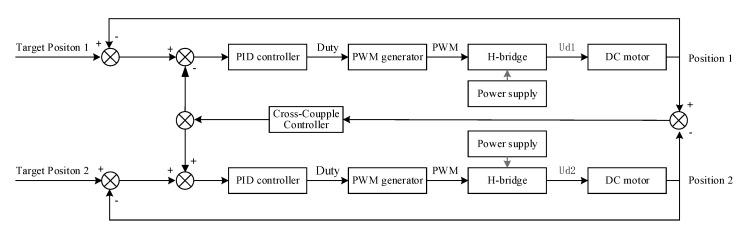
The control block diagram of the height adjustable desk system.

There are two motors in the mentioned height adjustable desk, for dual motor position synchronization problem, a cross-coupling control strategy is introduced to solve the dual-motor synchronization problem. The position bias between the two motors uses a cross-coupling control strategy based on PID (Proportion Integration Differentiation) control to compensate for the synchronous position error. The dual-motor position control method of the height adjustable desk system is shown in Figure 1. In the block diagram, for each motor, the error between the target position and real position is the input of the position PID controller. The PID controller is used to regulate the duty cycle of the PWM generator. The trigger signal PWM of power generator drive H-bridge to generate the armature voltage $U_{d}$, which is used to drive each motor. For dual-motor, there is a bias between real position 1 and 2. The bias as the cross-coupling controller compensates and diminishes the synchronous position error.

## 3. The Steady-State Kalman Filter Based Pinch Torque Estimation

In this section, pinch torque estimation based on the steady-state Kalman filter is presented. At the beginning of pinch torque estimation, pinch state variables should be selected correctly. Then, the Kalman filter is introduced. Besides, for linear time-invariant systems, the steady-state Kalman filter can replace with traditional Kalman filter to save computational resources. Finally, the flow of pinch detection based on the Kalman filter is presented.

### 3.1. Selecting State Variable for Pinch Detection

In the height adjustable desk system, there are so many state variables that can be selected as pinch detection state variables to detect pinch condition, such as armature current, current rate, motor angular velocity, angular velocity rate, motor torque, and torque rate. Torque and torque rate are less sensitive to the motor parameter. Moreover, it is more straightforward to select motor torque as a pinch detection state variable to detect pinch conditions. However, torque cannot be measured directly. To solve the above problem, state estimation with the Kalman filter is used to predict torque. The traditional torque estimation method is based on control torque as follows:

(4) $$\begin{array}{c} T_{c}=\left(U_{d}-K_{e}*\omega \right)\frac{K_{t}}{Ls+R} \end{array}$$

Because the electrical dynamics of the motor is much faster than the mechanical one, the control torque of the motor can be expressed as follows:

(5) $$\begin{array}{c} T_{c}\approx \frac{\left(U_{d}-K_{e}*\omega \right)}{R}*K_{t}=K_{t}*I_{d} \end{array}$$

**Figure 2:**
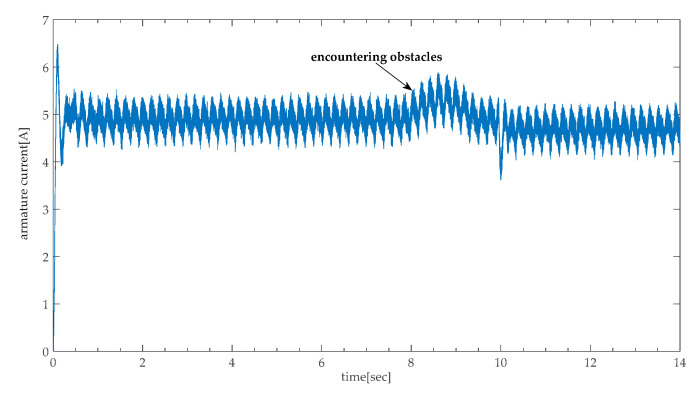
The curve of armature current when encountering obstacles.

From Equation (5), it can be seen that $T_{c}$ is proportional to armature current, which is equivalent to estimate armature current. Owing to the impact of dual motor synchronization, the amplitudes of oscillation of the armature current ripple are amplified, which makes it difficult to judge whether the height adjustable desk is encountering obstacles. The measured armature currents when encountering obstacles shown in Figure 2.

From Equation (2a–d), the following equation can be obtained:

(6) $$\begin{array}{c} T_{l}=T_{load}+T_{p}=K_{t}*I_{d}-J*\dot{\omega }-B_{\mu }*\omega +\mu_{v} \end{array}$$

**Figure 3:**
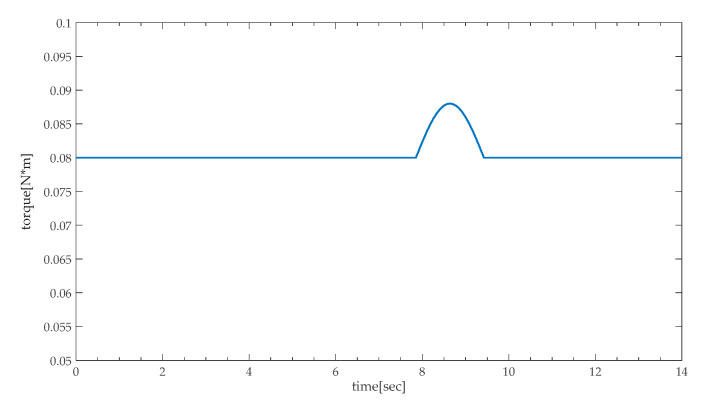
Load torque curve including initial load and encountering obstacles.

In Equation (6), $\;T_{load}$ is used to drive the initial load, which varies with the initial load. $\;T_{p}$ only appears when the height adjustable desk encounters obstacles. Thus, $T_{l}$ is selected as the pinch state variable. As shown in Figure 3, $T_{l}$ calculated by Equation (6) can present the change of load and the situation of encountering obstacles in height adjustable desk. Furthermore, it can be seen that the estimation of $T_{l}$ relies on the estimation of $I_{d}$ and ω. In the next section, the state-space model for pinch detection will be established based on the steady-state Kalman filter.

### 3.2. The Steady-State Kalman Filter Based Pinch Detection

In this section, pinch detection of the height adjustable desk based on the steady-state Kalman filter is designed to detect pinch state. From the above Section 3.1, $\;T_{l}$ is selected as the pinch state variable. According to [10,11], it is reasonable to augment the torque $T_{l}$ as an additional state and modeled by a random walk using the zero-mean white noise $\mu_{T}$ with the variance $Q_{T}$ as follows:

(7) $$\begin{array}{c} \dot{T_{l}}\left(t\right)=\;\mu_{T}\left(t\right) \end{array}$$

The state-space model can be expressed as follows:

(8) $$\begin{array}{c} \{\begin{array}{c} \dot{x}=Fx+Gu+w\\ y=Hx+v \end{array} \end{array}$$

where

$$\begin{array}{c} x={\left[I_{d}\;\omega \;T_{l}\;\right]}^{T},\;u=U_{d},\;y=I_{d},\;w={\left[0\;\mu_{v}\;\mu_{T}\right]}^{T}\\ F=\;\left[\begin{array}{ccc} -\frac{R}{L} & -\frac{K_{e}}{L} & 0\\ \frac{K_{t}}{J} & -\frac{B_{\mu }}{J} & -\frac{1}{J}\\ 0 & 0 & 0 \end{array}\right],\;G=\left[\begin{array}{c} \frac{1}{L}\\ 0\\ 0 \end{array}\right],\;H=\left[\begin{array}{c} 1\\ 0\\ 0 \end{array}\right] \end{array}$$

Let the process noise and the observation noise be uncorrelated. w(k) is the process noise that is assumed to be drawn from a zero-mean multivariate normal distribution with covariance Q(k), which $\mu_{v}\left(k\right)$ meets the Gaussian white noise with zero-mean and variance $q_{v}$, and $\mu_{T}\left(k\right)$ the Gaussian white noise with zero-mean and variance $q_{T}$. Similarly, the observation noise v(k) meets the Gaussian white noise with zero-mean and variance R(k).

Before the pinch observer design, it is necessary to prove the observability of (8), the judgment matrix of the observability is presented as follows:

(9) $$\begin{array}{c} rank=\left[\begin{array}{c} H\\ HF\\ HF^{2} \end{array}\right]=\left[\begin{array}{ccc} 1 & 0 & 0\\ -\frac{R}{L} & -\frac{K_{e}}{L} & 0\\ X_{1} & X_{2} & \frac{K_{e}}{L*J} \end{array}\right] \end{array}$$

where

$$X_{1}=\frac{R^{2}}{L^{2}}-\frac{K_{e}*K_{t}}{L*J},\;X_{2}=\frac{K_{e}*R}{L^{2}}+\frac{B_{\mu }*K_{e}}{L*J}$$

It is evident that the rank is 3, which means that the state-space Equation (8) can be observed. Thus, the armature current is suitable to be estimated and is used to estimate state variable $T_{l}$.

To facilitate the above design in discrete time, it is necessary to discretize the state-space model. Using a sampling period $T_{s}$, the state-space model can be rewritten as follows:

(10) $$\begin{array}{c} \begin{array}{c} x\left(k+1\right)=\Phi x\left(k\right)+\Gamma u\left(k\right)+w\left(k\right)\\ y\left(k\right)=Hx\left(k\right)+v\left(k\right) \end{array} \end{array}$$

where

$$\Phi =e^{FT_{s}},\;\Gamma =\left(e^{FT_{s}}-I\right)F^{-1}G$$

For simplification to be achieved, using the backward difference to make some approximation, they can be written as follows:

$$\begin{array}{c} \Phi =e^{FT_{s}}\approx FT_{s}+I\\ \Gamma =\left(e^{FT_{s}}-I\right)F^{-1}G\approx GT_{s} \end{array}$$

Then, Equation (10) can be expressed as follows:

(11) $$\left[\begin{array}{c} I_{d}\left(k+1\right)\\ \omega \;\left(k+1\right)\\ T_{l}\;\left(k+1\right) \end{array}\right]=\left[\begin{array}{ccc} 1-\frac{R}{L}*T_{s}\; & -K_{e}*\frac{T_{s}}{L} & 0\\ \frac{K_{t}*T_{s}}{J} & 1-\frac{B}{J}*T_{s} & -\frac{T_{s}}{J}\\ 0 & 0 & 1 \end{array}\right]*\left[\begin{array}{c} I_{d}\left(k\right)\\ \omega \;\left(k\right)\\ T_{l}\;\left(k\right) \end{array}\right]+\left[\begin{array}{c} \frac{T_{s}}{L}\\ 0\\ 0 \end{array}\right]*U_{d}+\left[\begin{array}{c} 0\\ \mu_{v}\\ \mu_{T} \end{array}\right]*T_{s}$$

After discretization is completed, the Kalman filter is introduced to modify and update the state variables. It is generally known that useful information is always affected by white noise in a real physical system. In this case, the Kalman filter is designed to minimize the variance of the estimation error. The standard Kalman filter consists of two parts: the predict step and update step.

For linear time-invariant systems (system matrices are not varying with time), the posteriori estimation error covariance matrix P(k|k) and the prior estimation error covariance matrix P(k|k−1) will converge towards steady-state values, which can be pre-calculated. Taking account of the real-time implementation issue, the steady-state Kalman filter gain is used. The steady-state estimation error covariance matrix $P_{\infty }$ holds the following discrete-time algebraic Riccati equation:

(12) $$\begin{array}{c} P_{\infty }=\Phi \left[P_{\infty }-P_{\infty }H^{T}{\left(HP_{\infty }H^{T}+R\right)}^{-1}HP_{\infty }\right]\Phi^{T}+Q \end{array}$$

Then, K(k) can be replaced with $K_{\infty }$ holding the following equation:

(13) $$\begin{array}{c} K_{\infty }=P_{\infty }H{\left(HP_{\infty }H^{T}+R\right)}^{-1} \end{array}$$

Thus, the above five equations about the Kalman filter can be simplified into two equations:

(14a) $$\begin{array}{c} \hat{x}\left(k|k-1\right)=\Phi \left(k\right)\hat{x}\left(k-1|k-1\right)+\Gamma \left(k\right)u\left(k\right) \end{array}$$

(14b) $$\begin{array}{c} \hat{x}\left(k|k\right)=\hat{x}\left(k|k-1\right)+K_{\infty }\left(y\left(k\right)-H\left(k\right)\hat{x}\left(k|k-1\right)\right) \end{array}$$

## 4. Threshold Design and Judgment Criteria of Pinch Condition

**Figure 4:**
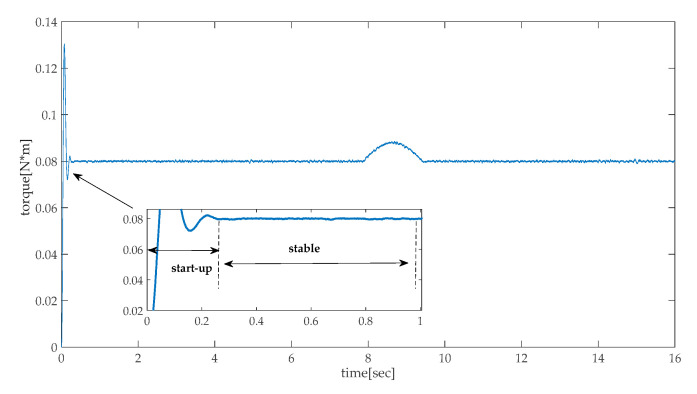
Estimated torque curve of the electric height adjustable desk’s running process.

**Figure 5:**
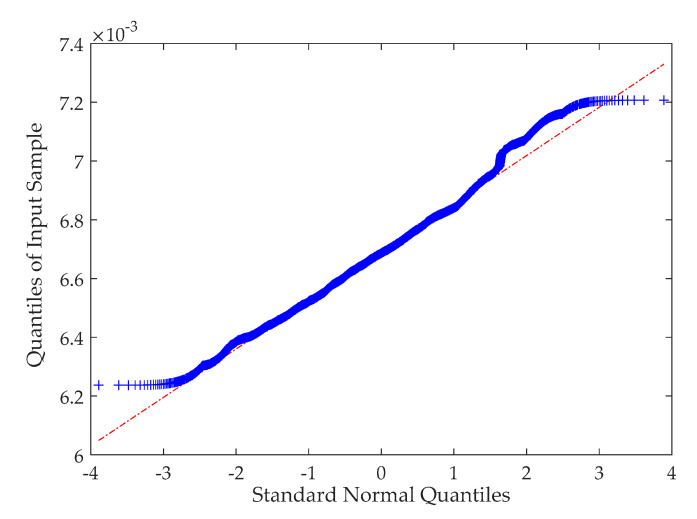
The quantile–quantile plot of the estimated torque data.

After the pinch torque is estimated, the remaining problem in pinch detection is to determine the threshold level of the torque. To observe the estimated torque data in Figure 4, avoiding the start-up phase of the motor, it can be seen that the torque has a certain distribution characteristic during the stable condition. The quantile–quantile plot is used to check the normality of the torque data. The result is shown in Figure 5. The quantile–quantile plot uses quantiles as the standard for evaluating the normality. If the data completely conform to the normal distribution, the scattered points form a straight line in the figure; the closer the scattered points are to the straight line, the better the normality. From Figure 5, the normality of the torque performs well. Thus, it is reasonable to use the confidence zone method to judge whether the obstacles are pinched [37].

To determine the torque’s confidence zone, firstly, obtaining the mean value $\mu_{T}$ of the torque in stable condition, m and n correspond to the end moment $t_{m}$ and the start moment $t_{n}$ of stable conditions, respectively.

(15) $$\begin{array}{c} \mu_{T}=\frac{1}{m-n+1}\overset{m}{\underset{j=n}{\sum }}T_{j}\; \end{array}$$

Then, to get the standard deviation of the torque in stable condition,

(16) $$\begin{array}{c} \sigma_{T}={\left[\frac{1}{m-n}\overset{m}{\underset{j=n}{\sum }}{\left(T_{j}-\mu_{T}\right)}^{2}\right]}^{\frac{1}{2}}\; \end{array}$$

According to the related knowledge of the normal distribution, the 99.73% confidence zone of the torque can be expressed as follows:

(17) $$\begin{array}{c} \Psi_{T}=\left[\mu_{T}-3*\sigma_{T},\mu_{T}+3*\sigma_{T}\right]\; \end{array}$$

$\Psi_{T}$ probably describes the range of possible changes of the torque when the height adjustable desk is on stable condition. In the case of the stable condition of the height adjustable desk, it is almost sure that the motor torque value will not exceed the confidence zone. Thus, the prescribed threshold can be set as $T_{th}$.

(18) $$\begin{array}{c} T_{th}=\mu_{T}+3*\sigma_{T}\; \end{array}$$

On the other hand, because the plant noise and observer noise exist during the height adjustable desk operation, if the prescribed threshold is compared with the estimated torque, there will be a misjudgment of pinch condition. Using the method of sliding root mean square with the estimated torque, the sliding root mean square can be expressed as follows:

(19) $$\begin{array}{c} T_{M}=\frac{1}{l_{m}}\sqrt{\overset{M}{\underset{j=M-l_{m}+1}{\sum }}T_{j}{}^{2}}\; \end{array}$$

$l_{m}$ is the window length value of the sliding, which can be determined according to the actual situation. In our application, $l_{m}\;$= 16.

Let the smoothed torque $T_{M}$ compare with the prescribed threshold $T_{th}$, when $T_{M}$ is greater than $T_{th}$, the anti-pinch function of the height adjustable desk starts, which reverses the motor to release obstacles.

## 5. Pinch Detection Experiments

In this section, an electric height adjustable desk platform is given. Then, the dual-motor position synchronization algorithm is verified in MATLAB, which is consistent with the actual control method, as shown in Figure 1. On the basis of dual-motor synchronization, the pinch detection method proposed in this paper is verified with MATLAB. The simulation is conducted under a slight and heavy load situation. In addition, the actual experiment is implemented to validate the performance of the height adjustable desk pinch detection algorithm proposed in this paper.

### 5.1. Simulation Conditions

To validate the performance of the proposed method, the simulations are given with MATLAB. Considering the plant noise caused by the power supply and PWM in practice, and the measurement noise v is introduced into the simulations, where the plant noise w and the measurement noise v conform to a zero-mean, with a Gaussian distribution with variance Q and variance R, respectively. They can be replaced by uncorrelated white noise. The motor parameters are obtained by the recursive least squares (RLS) in Table 1.

**Figure 6:**
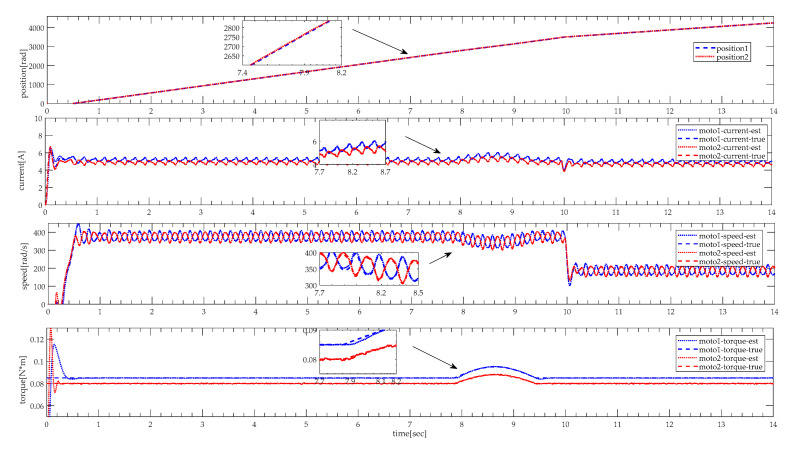
Status information under dual-motor position synchronization.

Owing to the coupling synchronization of two motors, there are mutual influences on their speed and armature current, which makes it hard to detect the pinch condition using them as pinch detection variables. Before the pinch detection, it is necessary to verify the synchronization between the two motors. As shown in Figure 6, there is a difference in the initial load of the two motors. Under the effect of cross-coupling control, dual motors achieve position synchronization. It can be seen that the torque increases distinctly when the height adjustable desk pinching obstacles, but the speed and current reflect the changes indirectly. That consolidates and verifies the accuracy of selecting the torque as a pinch detection state variable. In Figure 6, ‘position 1′ and ‘position 2′ represent the position of the two motors. ‘est’ and ‘true’ represent estimation results and simulation results of the model calculation mixed with the system noise, respectively. 

On the basis of the position synchronization of dual motors, in order to validate the performance of the height adjustable desk pinch detection algorithm proposed in this paper, the simulation experiments are implemented consisting of slight and heavy loads. According to the standards of the electric lifting desk industry, the pinch force must not exceed 100 N. In practice, the screw lead L of the height adjustable desk is generally 20 mm. The transmission efficiency η is 90%. The gear ratio i is 44:1. The relationship between screw thrust F and torque can be expressed as follows:

(20) $$\begin{array}{c} F*L=2*\pi *torque*i*\eta \; \end{array}$$

In our simulation experiments, the max load is set as 80 kg, which needs 800 N to drive. According to the above relationship between screw thrust and torque, the needed torque is about 0.064 N·m. Thus, 100 N is equal to 0.008 N·m. The pinching force can be simulated as follows:

(21) $$\begin{array}{c} \;F_{P}=0.008sin\left(\pi *t\right)\;t\in \left[2.5\pi ,3\pi \right] \end{array}$$

**Figure 7:**
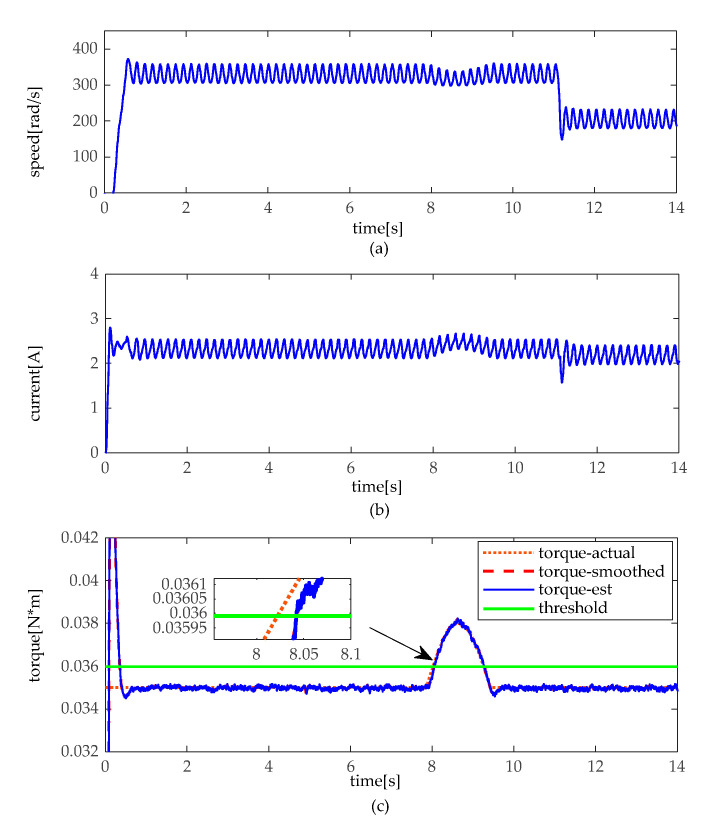
Pinch detection results of moto 1 in heavy load. (**a**) angular velocity. (**b**) armature current (**c**) pinch detection with torque.

**Figure 8:**
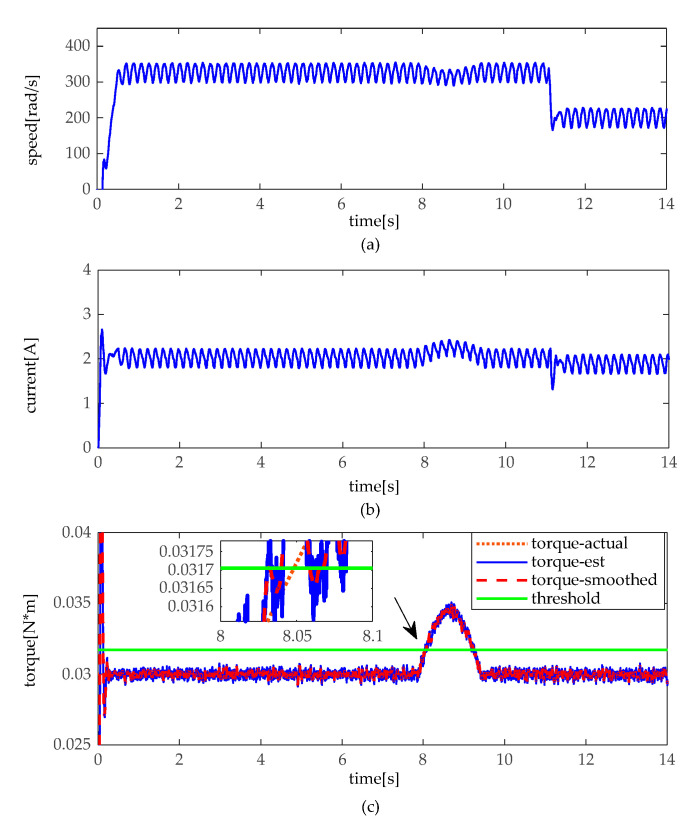
Pinch detection results of moto 2 in heavy load. (**a**) angular velocity. (**b**) armature current (**c**) pinch detection with torque.

**Figure 9:**
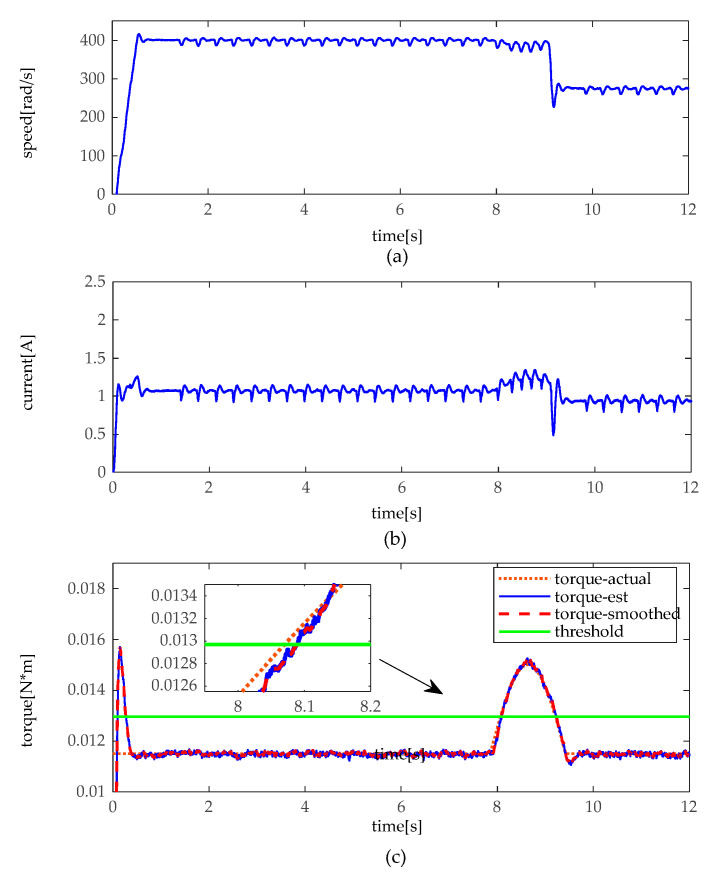
Pinch detection results of moto 1 in slight load. (**a**) angular velocity. (**b**) armature current (**c**) pinch detection with torque.

**Figure 10:**
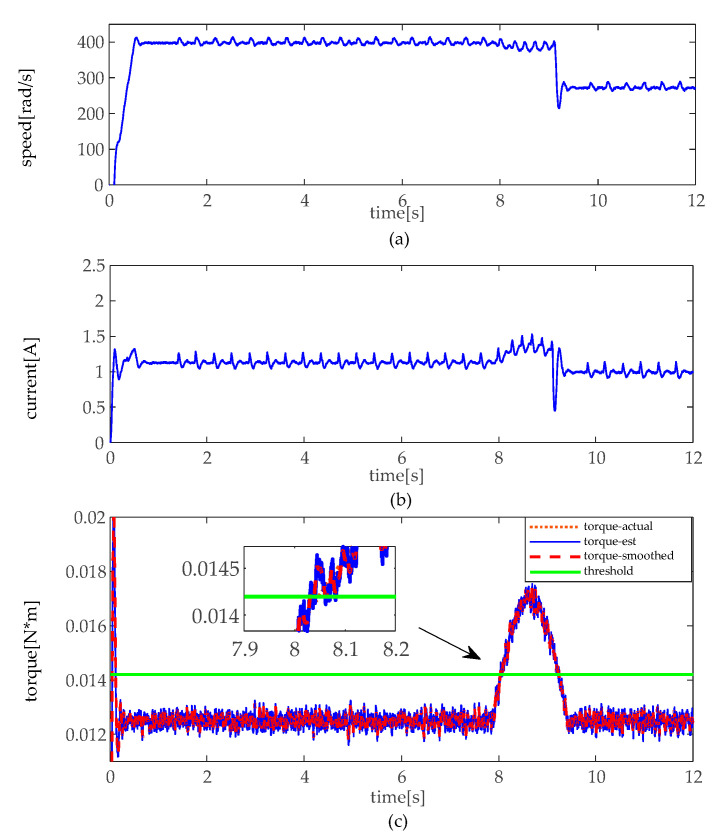
Pinch detection results of moto 2 in slight load. (**a**) angular velocity. (**b**) armature current (**c**) pinch detection with torque.

The simulation experiments are implemented under a slight and heavy load situation, which slight load is set under 30 kg (about 0.024 N·m), and heavy load of 80 kg (about 0.064 N·m). In practice, there is a different load to drive between moto 1 and moto 2. Thus, there exists a bias in loads. In the heavy load situation, the angular velocity, the armature current, and the torque of motor 1 and 2 are illustrated in Figure 7 and Figure 8. As shown in Figure 7 and Figure 8, when the height adjustable desk pinches the obstacles in heavy load, angular velocity slightly decelerates, and the armature current slightly increases as well. However, it can be seen that torque distinctly increases, and torque does not fluctuate like the angular velocity and armature current. In contrast, as shown in Figure 9 and Figure 10, when the obstacles are pinched by the height adjustable desk in a slight load, the angular velocity also slightly decelerates, but the armature current and torque change more obviously, armature current abruptly increases, and torque sharply rises. The simulation results show that the pinch detection algorithm can detect the pinch state regardless of a heavy load or light load.

### 5.2. Experimental Verification

**Figure 11:**
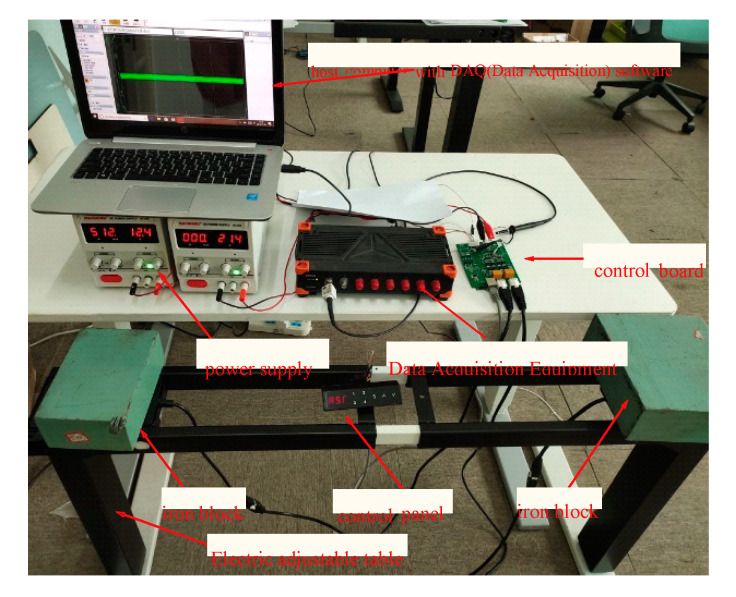
Electric adjustable desk.

**Figure 12:**
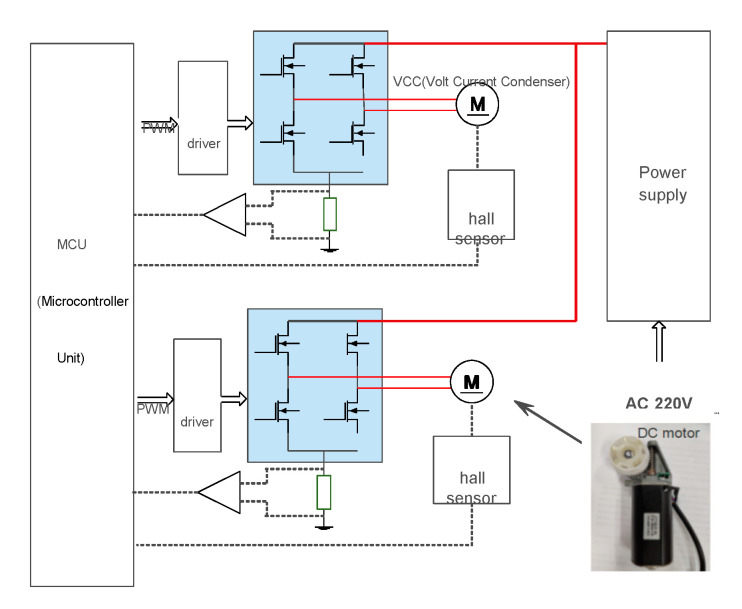
The control system structure.

The electric height adjustable desk test platform and control system structure are adopted in this paper to validate the performance of the proposed method, as shown in Figure 11 and Figure 12. In this platform, a DC power is supplied to the whole platform. The SIRIUS data acquisition instrument collects the motor armature currents and armature voltages with a 20 kHz sampling rate. The collected data observed by the SIRIUS are stored on the host computer. These collected data are used to estimate the torque. Two hall sensors are used to measure the height of the desk. Two sampling resistors are used to detect motor armature current. The STM32F103C8T6 produced by S.T. is selected as the control chip. The electric height adjustable desk is equipped with a separate lifting DC motor at each leg. Though a special motor drive chip, the control signal from STM32 is used to control the electric lifting desk movement.

**Figure 13:**
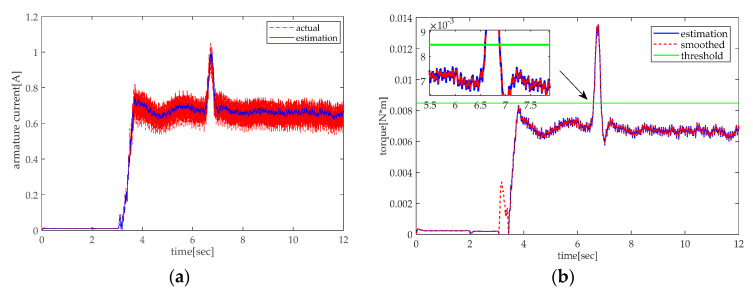
Pinch detection of the rising experiment: (**a**) armature current and (**b**) pinch detection with torque.

**Figure 14:**
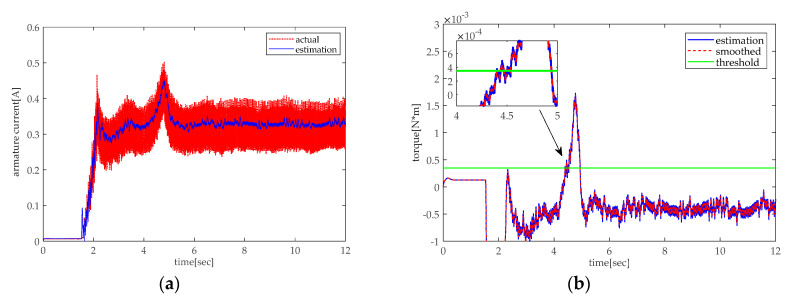
Pinch detection of the lowering experiment: (**a**) armature current and (**b**) pinch detection with torque.

In practice, the experiments are conducted in two situations, rising and lowering. As shown in Figure 13, there is an abrupt change of armature current and torque during the rising of the height adjustable desk when the desk pinches the obstacle. Figure 13b shows that the smoothed torque is greater than the threshold at about 6.6 s, which can trigger the motor’s retracting to release the obstacle. As shown in Figure 14, the torque effect will be less than 0 during the lowering of the desk owing to the effect of gravity. However, when the obstacle appears, there will be positive torque. The above simulation and experiment indicate that the pinch detection algorithm proposed in this paper can successfully detect the pinch condition.

## 6. Conclusions

A new algorithm based on the model reference Kalman torque prediction algorithm combines with the sliding root mean square (SRMS) is proposed to conduct the pinch detection of the electric height adjustable desk. To implement pinch detection, the system model is established by DC motor dynamics. Considering the position synchronization of the dual-motor, the cross-coupling control strategy is introduced into the system model. To eliminate the influence of dual motor synchronization and noise from PWM, a new pinch state variable for pinch detection is proposed and compared with the traditional pinch state variable. To save computational cost in MCU (Microcontroller Unit), the steady-state Kalman filter is introduced to estimate the pinch state variable. To improve the performance of the steady-state Kalman filter, the motor parameter is obtained by RLS. To judge pinch state, the sliding root means square method combined with the confidence zone is used to determine the dynamic threshold.

The simulations and experiments show that the electric height adjustable desk pinch detection method can successfully detect the pinch condition in different situations. Moreover, the proposed pinch detection algorithm can recognize pinch state under different loads. The proposed pinch detection method will improve the reliability and safety of the current electric adjustable desk anti-pinch function.

## 7. Patents

1. China Patent Application, CN ***201910508440.0***. A kind of electric adjustable desk resistance detection method and control device.
2. China Patent Application, CN ***201811374738.9***. Gesture recognition and position adjustment device for the electric adjustable desk.

## Figures and Tables

**Table 1:** Motor parameters.

Symbols	Values	Unit
Power supply	12	V
L	0.0709	H
R	1.17	Ω
$K_{e}$	0.0178	V/rad/s
$K_{t}$	0.0178	N·m/A
J	4.0 × 10^−6^	Kg·m^2^
$B_{\mu }$	1.9 × 10^−5^	Kg·m^2^

## Nomenclature

L	Armature inductance
$I_{d}$	Armature current
R	Armature resistance
$U_{d}$	Armature voltage
$E_{a}\;$	Motor back electromotive force
$K_{e}$	Back electromotive force constant
$K_{t}$	Torque constant
ω	Angular velocity
J	Moment of inertia
$B_{\mu }$	Viscous friction constant
$T_{c}$	Control torque
$T_{load}$	Load torque
$T_{p}$	Pinch torque
$T_{\mu }$	Friction torque
$\mu_{v}$	Vibration torque
A,F, and Φ	Coefficient matrix in the continuous system and discrete system, respectively
B,G, and Γ	Input coupling matrix in the continuous system and the discrete system, respectively
C,H	Observation matrix in the continuous system and the discrete system, respectively
w	Plant noise
v	Observation noise
u	System input
x,x(k)	State variables in the continuous system and the discrete system, respectively
y,y(k)	Observation variables in the continuous system and the discrete system, respectively
$T_{s}$	Sample time
P(k|k−1)	Covariance matrix of prior estimation.
P(k|k)	Covariance matrix of posterior estimation
K(k)	Kalman gain
Q	Plant noise variance
R	Observation noise variance
·	Denotes derivative
^	Denotes an estimated value
